# How do paramedics learn and maintain the skill of tracheal intubation? A rapid evidence review

**DOI:** 10.29045/14784726.2018.09.3.2.7

**Published:** 2018-09-01

**Authors:** Richard Pilbery

**Affiliations:** Yorkshire Ambulance Service NHS Trust

**Keywords:** intubation, paramedic, skill acquisition, skill retention

## Abstract

**Introduction:**

Endotracheal intubation has been considered a core skill for all paramedics since the inception of the profession in the 1970s, and continues to be taught within the majority of pre-registration paramedic training programmes. However, the standards of both training and assessment of competence in intubation vary considerably between institutions; this has been compounded by reduced opportunities for supervised clinical practice within the operating theatre environment.

The College of Paramedics’ Airway Working Group commissioned a rapid evidence review, to inform a consensus statement on paramedic intubation, with the research question: How do paramedics learn and maintain the skill of tracheal intubation?

**Methods:**

Rapid evidence reviews are literature reviews that use methods to accelerate or streamline the traditional systematic review process. Randomised controlled trials, quasi-randomised controlled trials, prospective and retrospective observational studies, systematic reviews and qualitative studies, published from 1970 onwards, were all eligible for inclusion. The search was restricted to paramedics/paramedic students and learning/maintaining the skill of tracheal intubation.

**Results:**

A comprehensive search of CINAHL, MEDLINE and Google Scholar was undertaken. Ten papers were classed as sufficiently relevant for inclusion. They identified that there is no clear definition of a paramedic having ’learnt’ the skill of intubation. Suggested measures include first-pass success of 90% for pre-hospital intubation, or a range of measures, such as intubation success and complication rates, laryngoscopy technique and decision-making. Intubation training should use a range of modalities, including didactic lectures, videos and practical sessions on multiple types of airway manikins. Supervision by experienced faculty is required.

Little is known about how paramedics maintain their skill in intubation, given the lack of clinical opportunity. Yearly skills retraining can help, and can be enhanced by demonstrations/lectures from experienced faculty.

**Conclusion:**

Further research is needed to understand how paramedics maintain their skill in intubation, given the limited opportunities to use the skill in a clinical setting and lack of opportunities with UK ambulance services for retraining.

## Background

Endotracheal intubation (ETI) has been considered a core skill for all paramedics since the inception of the profession in the 1970s, and continues to be taught within the majority of pre-registration paramedic training programmes. In recent years, the practice of intubation by paramedics has been widely debated and its continued use questioned. The increasing availability of supraglottic airway devices, guidance from the Joint Royal Colleges Ambulance Liaison Committee and Resuscitation Council shifting the emphasis to the primary use of such devices rather than intubation in most patients, and limiting the opportunity for paramedics to intubate in clinical practice have all contributed to the debate (Younger, Pilbery, & Lethbridge, 2016).

The transition from vocational Institute of Health and Care Development national paramedic training to preregistration programmes delivered within Higher Education Institutions has resulted in the standards of both training and assessment of competence in intubation varying considerably between institutions; this has been compounded by reduced opportunities for supervised clinical practice within the operating theatre environment.

### Research question

This rapid evidence review (RER) aimed to inform the College of Paramedics’ Airway Working Group as they revise the previous consensus statement on paramedic intubation (College of Paramedics, 2008). The research question of this RER was: How do paramedics learn and maintain the skill of tracheal intubation?

### Objectives

The objectives of the RER are to:

- evaluate literature from 1970 (the approximate time that the role of ’paramedic’ first came into existence) to the present that examines how paramedics learn the skill of intubation and how competence in the skill is maintained; and
- present the findings from the review to assist the Airway Working Group in developing and publishing a revised consensus relating to paramedic intubation.

## Methods

### Rapid evidence reviews

Due to constraints on cost and time, a systematic review could not be undertaken. Instead, an RER based on the methodology outlined by Collins, Coughlin, Miller, and Kirk (2016) was utilised. RERs (also referred to as rapid evidence assessments or rapid reviews) are literature reviews that use methods to accelerate or streamline the traditional systematic review process (Ganann, Ciliska, & Thomas, 2010). As such, they are typically completed in compressed timeframes when compared to a systematic review.

### Inclusion and exclusion criteria

In order to identify relevant studies that address the research question, the PIOS (participants, interventions, outcomes, studies) acronym was used (Table 1). Readers familiar with PICOS will note the omission of the ’C’ for comparator. This parameter was not relevant to the research question in this RER and so was not included.

**Table 1.** Summary of inclusion and exclusion criteria.

PIOS	Inclusion criteria	Exclusion criteria
**Participants**	Paramedics* and/or student paramedics	Other allied health-professionals, nurses and doctors
**Interventions**	Tracheal intubation	Other airway management techniques
**Outcomes**	Learning and maintenance of skill	Morbidity/mortality benefit of skill
**Studies**	Randomised controlled trials, quasi-randomised controlled trials, prospective and retrospective observational studies, systematic reviews and qualitative studies	Editorials, position statements, letters, literature reviews, case reports and consensus statements

Studies including paramedics and other healthcare professionals could be included if paramedic data could be separated.

#### Participants

The search was restricted to paramedics and/or paramedic students in an attempt to ensure that the participants were as homogeneous as possible. It is accepted that there are variations in the advanced airway management of paramedics from different countries and, in this search, potentially across the decades. However, it is assumed for this review that paramedics by this definition are a more homogeneous group than if nurse anaesthetists or doctors had been included.

#### Interventions

This was restricted to tracheal intubation, although the method of intubation (e.g. direct or video laryngoscopy) was not restricted in the search. However, studies that only addressed whether direct or video laryngoscopy was the best method of intubation, for example, were not eligible. No comparator was specified for this review.

#### Outcomes

This review focused on the acquisition and maintenance of the skill of intubation, so studies that purely aimed to address questions about whether paramedics *should* intubate or not were not eligible for inclusion.

#### Studies

Randomised controlled trials (RCTs), quasi-randomised controlled trials, prospective and retrospective observational studies, systematic reviews and qualitative studies, published from 1970 onwards, were all eligible for inclusion. There was no restriction on language, but results were limited to research on humans. Editorials, position statements, letters, non-systematic literature reviews, case reports and consensus statements were not eligible.

### Search strategy

Both CINAHL and MEDLINE were used for the literature search, with grey literature searched via Google Scholar (the first 100 results from this search were included). In addition, subject-matter experts from the College of Paramedics’ Airway Working Group were consulted about relevant papers they were aware of. An initial scoping search to identify appropriate keywords and MESH headings was undertaken by librarians at the University of Hertfordshire. The final CINAHL/MEDLINE literature search query and Google Scholar search were run on 23 December 2017. Full details of both can be found in Supplementary 1.

### Study selection

A ’first-pass’ of the search results was conducted by a single individual (RP) who screened the title and abstract against the inclusion/exclusion criteria to determine whether it might be suitable for inclusion. Once this was completed, the full-texts of papers that had made it through the first-pass process were obtained and reviewed (’second-pass’). Those that met the inclusion criteria were put forward for inclusion in the review. In addition, the references sections of the full-text papers which were excluded at the second-pass stage were screened for potentially relevant studies to inform the review.

### Critical appraisal

Papers that successfully made it past the second-pass process were critically appraised in two stages. The first stage of the critical appraisal phase was to determine the relevancy of the paper in relation to the RER research question. A score of 1–3 was awarded for relevancy based on the population, intervention and outcome of the study, with 1 indicating low relevancy and 3 high.

The robustness of the evidence was determined by evaluating each paper against a list of criteria. Separate lists of criteria were utilised for quantitative interventional and observational studies, qualitative studies and systematic reviews. Each criterion was given a score of 1–3 (1 being the lowest), and from these an overall critical appraisal score of 1–3 was awarded, based on the most commonly awarded score for each criterion. *Only papers that scored more than 1 for relevancy and 1 for robustness were included in the final review synthesis.*

### Data synthesis

Once the final papers for inclusion in the RER had been selected, a narrative synthesis of the evidence was conducted. This consisted of:

1. describing the volume and characteristics of the evidence base;
2. utilising the synthesis to answer the research questions;
3. highlighting the implications of the findings; and
4. making recommendations for further research.

## Results

Based on the search strategy, 939 results were returned from the CINAHL/MEDLINE search and the first 100 results from the Google Scholar search were included. Following removal of duplicates, 1009 records remained for ’first-pass’ screening (review of title and abstract). ’Second-pass’ screening (review of full-text) was under taken for 77, which also identified a further eight papers. The full-text was retrieved for these papers and they were included in the ’second-pass’ screening process. Seventeen passed the second-pass screening, with 10 being classed as sufficiently relevant and robust to be included in this review (Figure 1). The complete list of first- and second-pass papers, including reason(s) for exclusion, can be found in Supplementary 2. Second-pass papers which were not sufficiently robust or relevant can be found in Supplementary 3.

**Figure:**
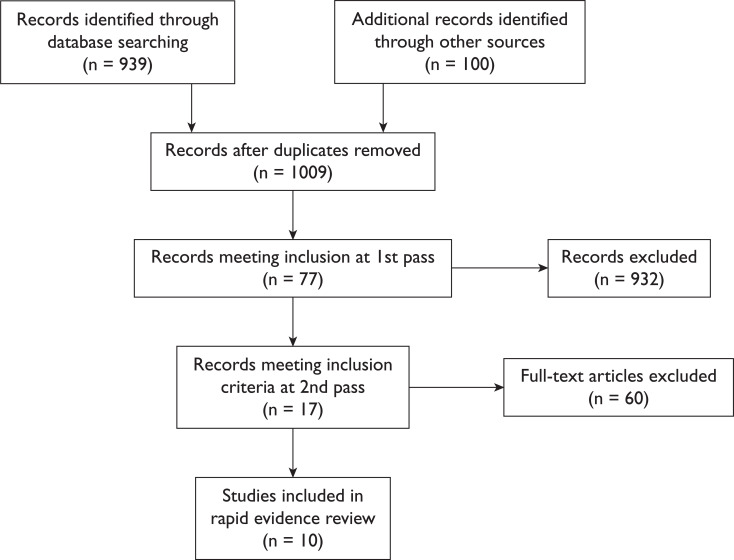
Figure 1. PRISMA diagram of literature search.

### Description of included studies

The final 10 studies included in this review consist of eight papers that examine how paramedics acquire the skill of intubation, one paper that addressed intubation skill maintenance by paramedics and one study that attempted to address both. Papers came from several countries: six from the United States, and one each from the UK, Canada, Australia and Japan. Publication dates for papers varied, with six published 10–20 years ago and four published within the last 10 years. Critical appraisal scores were fairly evenly split, with five papers scoring two, and five scoring three. All included papers scored a two for relevancy.

Most papers (seven) were interventional in design, with two of these being RCTs. The remainder were observational. Studies that examined intubation attempts by paramedics as an outcome included a mixture of live intubation assessments, either in an operating theatre setting or, in some cases, out-of-hospital, and manikin-only assessments. A summary of study participants, outcomes and results can be seen in Table 2. The critical appraisals of each of the second-pass papers can be found in Supplementary 4.

**Table 2.** Summary of study participants, outcomes and results.

Citation, data collection period and country	Study type	Participant details	Outcomes/outcome measures	Results	CAS	REL	Comment
Deakin et al. (2009) May 2008 UK	Retrospective, observational (PRF, review) Prospective, observational (survey)	269 paramedics with a documented intubation attempt. Median number of intubations during study period is 1 (range 0–11). 15 UK ambulance services responded to survey.	PRF review: intubation success rate, intubations per paramedic per annum, intubation attempts. LMA insertion also included. Survey: LMA use by technicians/paramedics, initial and ongoing training for intubation and LMA.	128/269 paramedics (47.6%) had undertaken no intubation and 204 (75.8%) had undertaken one intubation or fewer in the 12-month study period. First-pass success occurred in 320/394 (81.2%) of attempts (no data recorded in 45 PRFs). 15 ambulance services responded to survey. 13 required intubations to be performed in theatre. 10 required 25 intubations, 2 required 20 (one of these had a caveat that this was acceptable if assessed as competent), 1 required 13 (if competent), 1 required 10, 1 required 5. For ongoing training, 3 services conducted annual manikin assessment, 2 conducted a mannikin assessment every 2 years and 1 sent paramedics to spend 1 day in theatre if number of intubations deemed inadequate.	2	2	Survey undertaken during transition to higher education. Most likely historic picture of previous, vocational-style requirements
Hall et al. (2005) May–Dec 2003 Canada	Prospective, interventional (RCT)	36 second-year paramedic students with no prior intubation training or experience. 540 patients (270 in each group), mean age 43.0 years (range 3–88 years) in the SIM group and 44.1 years (range 15–87 years) in the OT group (p = 0.48). 12 patients under 18 years in the SIM group and 7 in the OT group (p = 0.24). The study groups were well matched for predictors of airway difficulty including: BMI, dentures, Mallampati scores, thyromental distance, paralytic use and overall airway difficulty.	Overall intubation success rate. Intubation success, first attempt success, complications incl. dental trauma, airway bleeding, oxygen desaturation <85%, arrhythmias, oesophageal intubation.	Overall success rate 87.8% in SIM-only group and 84.8% in the OT group. First attempt success rate 84.4% and 80% (SIM and OT groups respectively). Neither statistically significant. 11/18 students in the SIM group ? 13 successful intubations. 12/18 students in the OT group ? 12 successful intubations. The mean time to successful intubation was 47.2 seconds in the SIM group and 43.0 in the OT group, with a difference of 4.2 (95% CI = ?0.5 to 8.8). Skill acquisition during the process of testing did not occur in either group, because the overall success rates were consistent throughout the 15 test intubations. Test intubations were interrupted for patient safety in 13 of 540 test intubations (2.4%). 8 of 270 test intubations (2.9%) in the SIM group and 5 of 270 (1.9%) in the OT group were interrupted (p = 0.57). Reasons included airway difficulty (n = 5), ventricular arrhythmia during laryngoscopy (n = 1), anaesthetist unaware of study protocol (n = 1), student’s request (n = 1) and unspecified (n = 5). The mean time from training to testing was 7.5 weeks in the SIM group and 7.0 weeks in the OT group (p = 0.99). There was no difference between groups for the overall intubation success rate in students with longer delays between training and testing (p = 0.31)	3	2	N/A
Johnston et al. (2006) June–Sept 2005 USA	Prospective, observational	161 programme directors of CAAHEP accredited paramedic training programmes.	Opportunity for intubation by paramedic students. Average training hours per student. Average intubation attempts, student support, graduate fulfilment of national recommendation of 5 intubations, access to operating theatres over past 2–3 years.	Median time in theatres 17–32 hours. Half of programmes provided < 16 hours per student. Median intubation attempts 6–10 per student. 58% respondents reported increased competition for operating theatre placements to practise intubation. Increasing use of LMAs and medical-legal concerns highlighted in free text. Reduction in access identified by 52 programmes (32%) and 56 (35%) expected operating theatre placements to reduce in next 2–3 years.	2	2	161/192 (85%) completed surveys returned
Levitan et al. (2001) 1995–1998 USA	Prospective, interventional (historic controls)	Paramedic students (no other demographic data provided).	Intubation success, number of intubations per student. Differences between groups in terms of participant age, gender and level of education.	113 students, comprising 4 years of paramedic classes (1995–1998, i.e. control group), performed 783 laryngoscope insertions. The mean intubation success rate was 46.7% (95% CI, 42.2–51.3%, SD ± 24.7%). The range of laryngoscope insertions per student was 1–15 (mean 6.99, mode 6). In the video (intervention) group (paramedic classes 1998–1999), 36 students performed 102 laryngoscopies, with a mean individual success rate of 88.1% (95% CI, 79.6–96.5%, SD ± 25.9%). The range of insertions was 1–10 (mean 2.8, mode 3). Comparing the traditional group with the video group, the difference in success rates was statistically significant (P ? 0.0001; 46.7% vs. 88.1%, difference 41.4%, 95% CI, 31.1%–50.7%). The video and traditional groups did not differ in terms of age (25.0 vs. 26.1, P = 0.48), male sex (65.8% vs. 52.6%, P = 0.147) or level of education (87.5% grade 12 vs. 86.8% grade 12, P = 0.375).	2	2	N/A
Plummer and Owen (2001) 1 year, date not specified (paper accepted May 2001) Australia	Prospective, interventional	115 students, mostly medical students (95), remainder critical care trainees and student paramedics (13).	Models of intubation success.	The rate of successful ETI increased from 6% on the first trial to approximately 80% after 15 trials. Trainees became familiar with an airway trainer after multiple trials, as demonstrated by a 50% decrease in the odds of successful ETI when starting on a new trainer. The learning model indicated that a trainee learns about as much from 1 successful ETI as from 12 (95% CI, 2–23) failed trials. The log of the number of intubation attempts correlated with intubation success (OR 6.8, 95% CI, 4.3–11). Paramedic students were significantly better than medical students. Choice of instructor did have a significant adverse effect on intubation success.	2	2	Owen and Plummer (2002) was not included in this review (was primarily a description of a new clinical simulation unit). Did describe some data from this study. Suggested paramedic students approached 100% success after 6 attempts
Toda et al. (2013) Jan 2005–Dec 2011 Japan	Prospective, interventional	32 paramedics, no details on selection. Patients: healthy surgical patients who required intubation as part of their anaesthetic management and who were: aged 20 years or older, ASA physical status class I or II and no evidence of a potentially difficult airway.	How much does the success rate of tracheal intubation by paramedics improve over the course of the 30 live experiences? How much is the frequency of complications possibly associated with tracheal intubation decrease?	32 paramedics attempted 1049 intubations. 4 attempts aborted. 1 due to no vocal cord visualisation, 1 due to tooth mobility and 2 because of dental damage during BVM ventilation. Only data for each trainee’s first 30 patients were used to avoid survival bias, giving 960 observations for analysis. Overall success rate increased from 71% to 87% (CI, 82–94%) after training on 30 patients. Used model to predict number of attempts required for 90% and 95% success: 31.5 (95% CI, 27.6–54.3) and 38.6 (CI, 31.2–76.9). Complications all minor. Overall complication rate decreased from 53% to 31% after 30 patients. No significant learning up to 13 experiences with fastest learning period around 19 intubations.	3	2	Unclear whether skillset of paramedics in Japan comparable with UK
Wang et al. (2005) May 1999–Dec 2003 USA	Retrospective, observational	891 students from 60 paramedics programmes in USA, 802 attempted a total of 7635 ETIs. No ETIs were reported by 89 students. Only first 30 intubation attempts included, leaving 7398 intubations for study inclusion. No student or patient demographics available.	Relationship between intubation success and cumulative number of intubations.	Mean number of intubations per student 9.5 (median 7, IQR 4–12). Self-reported intubation success. Overall 87.5% (95% CI, 86.7–88.2), pre-hospital (n = 903) success 74.8% (71.9–77.6%). Learning curve for paramedics increased from 77.8% to 95.8% over 30 ETI procedures. When stratified by clinical setting, suggests more than 30 intubations required to achieve >90% success rate with pre-hospital intubations.	2	2	N/A
Warner et al. (2010) 3 years, no start date (paper submitted Jan 2009) USA	Retrospective, observational	56 paramedic students from Seattle Fire Department. No other details.	Primary outcome successful placement of an ETT in the trachea by the student, regardless of number of attempts. Secondary outcome. First-pass success rates for student ETI attempts in the pre-hospital setting.	56 paramedic students in 3 consecutive classes completed training and were included in study. 1616 intubations attempted (median 29 intubations per student), 706 intubations in operating theatres and 576 in pre-hospital setting. Pre-hospital intubation success 88% for all students, first-pass success 66%. Odds of intubation success increased by 1.097 for each successive patient (95% CI, 1.026–1.173). First-pass success OR 1.061 (1.014–1.109). Cumulative exposure to pre-hospital intubation most important factor in pre-hospital success. First-pass success of 90% requires more than 20 attempts.	3	2	N/A
Wong et al. (2011) No study period specified (paper accepted Aug 2010) USA	Prospective, interventional	51 paramedic students in their second month of training and 18 medical students, including 12 first-year, 3 second-year, and 3 fourth-year students.	Intubation success or failure and time to intubation.	Overall 88% ± 1% success rate. first-pass success 65–70%. Success rate improved with number of attempts (OR 1.06, 95% CI, 1.03–1.08). Paramedic students more likely to succeed than medical students. Students trained on the novel trainer in a static configuration were less likely to be successful at intubating compared to the group using the Laerdal airway trainer. A recent change in airway model reduced the odds of success to 70% of the odds without a change. However, practising laryngoscopy in a new airway model adjusted into 5 different configurations did not improve the odds of success over practising with only an airway trainer held in a fixed anatomy.	3	2	N/A
Youngquist et al. (2008) 24 months, date not specified (paper received April 2008)	Prospective, interventional	245 paramedics, 184 male, median years as paramedic 8 (IQR 4–13), 141 paramedics were parents, total runs and paediatric runs per 24hr reported, months since elapsed training median 13 (IQR 7–16). Demographics per group also included.	Self-reported confidence and anxiety performing BVM and ETI. Skills performance. Mean change between self-efficacy scores and skill performance.	Paramedics from low-call-volume areas reported lower baseline self-efficacy and derived larger increases with training, but also experienced the most decline between training events. Pass rates for BVM and ETI were 66% (139?211) and 42% (88?212), respectively. Overall cohort self-efficacy was maintained over the study period. In ordinal regression modelling, only the lecture and demonstration method was superior to control, with an OR of achieving higher scores of 2.5 (95% CI = 1.2–5.2) for BVM and 5.2 (95% CI, 2.4–11.2) for intubation. Poor performance with intubation but not BVM was associated with time elapsed since training (p = 0.01). Self-efficacy ratings were not predictive of skill performance.	3	2	N/A

ASA = American Society of Anesthesiologists; BMV = bag-valve-mask; CAAHEP = Commission on Accreditation of Allied Health Education Programmes; CAS = critical appraisal score; CI = confidence interval; ETI = endotracheal intubation; IQR = interquartile range; LMA = laryngeal mask airway; OR = odds ratio; OT = operating theatre; PRF = patient report form; RCT = randomised controlled trial; REL = relevance score; SIM = simulation.

#### Pre-hospital advanced airway management by ambulance technicians and paramedics: is clinical practice sufficient to maintain skills?

This paper by Deakin, King, and Thompson (2009) consisted of a retrospective patient report form (PRF) review and a prospective telephone survey of UK ambulance service training schools. This was undertaken with the aim of assessing current airway practice, in order to review whether the initial training and maintenance of airway skills provided an acceptable level of competence. The PRF review was conducted in a single ambulance service, and the telephone survey included 15 ambulance services in the UK, which is likely to be all of the services at the time of the study.

#### Human patient simulation is effective for teaching paramedic students endotracheal intubation

Hall et al. (2005) set out to test their hypothesis that simulator training was as effective as using live patients for teaching paramedic students to intubate. The study was an RCT involving 36 second-year paramedic students from a single training college in Canada who, following their standard airway training (20 hours didactic and video and 10 hours manikin training), were randomised into either a simulation-only group or traditional operating theatre placements. Students in the simulation-only group were subjected to a standardised curriculum including instruction of each basic step of intubation and repetition of the technique with various airways and case scenarios. The average student completed 50 intubations on the simulator with a range of 40–70, depending on each student’s proficiency. A specific number of intubations was not required during simulator training; rather, students were required to achieve excellent technique with advancing levels of airway difficulty. Difficult airways were achieved by using cervical spine immobilisation and using the simulator options of tongue swelling, oropharyngeal swelling and laryngospasm. Students in the operating theatre (control) group underwent the local standard of obtaining 15 intubation attempts in theatres, under the supervision of an anaesthetist.

Both groups were subsequently tested by attempting 15 intubations on patients in operating theatres as part of the study assessment. There were no formal patient inclusion/exclusion criteria specified. Patients were selected on a non-consecutive basis at the discretion of the anaesthetist.

The primary analysis (to determine overall intubation success and first attempt success rates) was performed using a generalised estimating equation (GEE) to account for the cluster structure in the design arising from the repeat assessments for each student. Demographic variables were compared using unpaired t-tests, chi-square tests and Fisher’s exact tests, depending on the nature of the demographic variables.

#### Limited opportunities for paramedic student endotracheal intubation training in the operating room

Johnston, Seitz, and Wang (2006) conducted a survey of 161 programme directors of accredited training programmes in the United States. They sought to ’characterise the nature of the operating theatre training provided to paramedic students for learning tracheal intubation’. An anonymous closed-question survey was sent out, although free-text comments were encouraged. Analysis was limited to descriptive statistics (medians and interquartile ranges (IQRs)). A total of 192 programme directors were contacted and 161 completed surveys were returned, a response rate of 85%.

#### Training with video imaging improves the initial intubation success rates of paramedic trainees in an operating room setting

Levitan, Goldman, Bryan, Shofer, and Herlich (2001) undertook a study to test the hypothesis that training with the use of videos of laryngoscopy from the point of view of the person using the laryngoscope, in addition to traditional didactic and manikin instruction, would improve success rates of paramedic students when intubating in operating theatres. In the intervention (video) group, students viewed a 26-minute video showing 15 laryngoscopies. Each student had to watch the video three times prior to commencing their operating theatre placement, in addition to the standard 42 hours of classroom instruction, consisting of didactic teaching and manikin practice that students traditionally undertook. Note that there were no specified inclusion/exclusion criteria. In total, 149 students provided data on 805 intubation attempts in operating theatres.

#### Learning endotracheal intubation in a clinical skills learning center: a quantitative study

Plummer and Owen (2001) based their study on the hypothesis that the process of learning to intubate airway trainers (manikins) may be subjected to quantitative analysis, and that such analysis would provide insight into how instruction could be further improved. A total of 115 students, in groups of 2–4, took part in a training session in a clinical simulation unit. Each session lasted 75–90 minutes and consisted of:

1. an intubation video;
2. equipment familiarisation;
3. a demonstration;
4. student attempts on an ’easy’ simulator;
5. feedback on technique;
6. repeat attempts with feedback until consistent performance;
7. exposure to different/difficult simulators/introducing alternative techniques and intubation aids.

Students typically had up to 17 intubation attempts during the session on a range of airway trainers (although, based on Owen and Plummer (2002), not all students were able to practise on all trainers).

#### Learning curve for paramedic endotracheal intubation and complications

The objective of Toda, Toda, and Arakawa’s (2013) study was to assess the efficacy of the intubation training programme for paramedics by addressing two questions: 1) How much does the success rate of tracheal intubation improve over the course of the 30 live intubation attempts? 2) How much does the frequency of complications possibly associated with intubation decrease? A total of 32 paramedics (with no previous intubation experience on live patients) were trained in tracheal intubation between January 2005 and December 2011. All trainees received instruction in the theoretical aspects of intubation through attending a standardised lecture, watching a video and then practising on a manikin. Study participants intubated patients chosen by their supervising anaesthetist and were allowed two attempts per patient. The anaesthetist recorded the number of attempts and any complications possibly associated with the intubation, including: hoarseness, sore throat, lip laceration, oral bleeding, gingival bleeding, lip bleeding, pharyngeal bleeding, tongue laceration, dental damage, lip swelling and tongue bleeding.

The authors constructed a generalised logistic regression model to determine the probability of success, including variables of initial and final success rates, learning speed and number of intubation attempts at which the success rate improved the most. A similar model was constructed for the probability of complications.

#### Defining the learning curve for paramedic student endotracheal intubation

Wang, Seitz, Hostler, and Yealy (2005) sought to determine whether paramedic student intubation success is associated with accumulated live intubation experience, adjusted for elapsed time since first intubation attempt and the clinical setting (i.e. operating theatre, emergency department, intensive care, other in-hospital or out-of-hospital). Data for the study were obtained from the clinical and procedural experience tracker, Fisdap, which at the time of the study was used by 175 paramedic programmes throughout North America. Students elected to allow the sharing of their anonymised data for research purposes.

Fixed-effects logistic regression was performed to model the learning curve for intubation while adjusting for multiple relevant covariates and to account for per-paramedic student clustering effects. Intubation success was modelled as the primary binary outcome. The key independent variable was cumulative number of intubation attempts. In addition, adjustments were made for clinical setting and elapsed days since first intubation attempt.

#### Paramedic training for proficient pre-hospital endotracheal intubation

Warner et al. (2010) hypothesised that the number of intubation attempts by paramedic students early in their training would be associated with a greater intubation success rate prior to graduation. They performed a retro-spective, observational analysis of intubation attempt data collected as part of a training programme for Seattle Fire Department paramedic trainees.

Students in this programme undertake a nine-month training programme and receive 2200 hours of training divided between 400 hours of lectures, 100 hours of laboratory work, 600 hours of hands-on clinical work, 800 hours of field internship and 300 hours of formal evaluation. Exposure to intubation begins with intensive manikin training supervised by paramedic instructors and coincides with lectures on airway management and skill laboratories. Students receive practical clinical experience in operating theatres where they are taught airway assessment, bag-mask ventilation, direct laryngoscopy and rescue techniques by anaesthetists. After completing a minimum of five successful intubations in adults in theatres, students are allowed to perform airway management in the field under the direct supervision of senior paramedics, but continue to acquire additional airway management experience in the operating room.

Pre-hospital airway management experience extends for approximately eight months. Students may attempt intubation in the emergency department with permission and direct supervision from attending physicians. Additional education in paediatric airway management is provided in theatres of Seattle Children’s Hospital under direction of faculty from the University of Washington School of Medicine Department and Anaesthesiology. Advanced training in surgical airway management is provided by the Director of Paramedic Training and the University of Washington Department of Surgery in simulation laboratories.

The data for analysis were collected as part of the quality assurance process for the paramedic training. Data on each student’s intubation attempt were described using measures of central tendency for continuous data and percentages for categorical data. Multi-variable logistic regression, using GEEs with robust variance estimators, was used to assess the effect of cumulative experience on paramedic student pre-hospital intubation success rates while adjusting for confounding variables. Cervical spine precautions in trauma, cardiac arrest and rapid-sequence induction were considered as potential confounders in the analysis. The relationship between pre-hospital intubation success rate and total number of intubation attempts was summarised by plotting the predicted success rate and 95% prediction intervals from the regression equation as a function of cumulative number of intubation attempts.

#### The effect of cross-training with adjustable airway model anatomies on laryngoscopy skill transfer

Wong et al. (2011) tested the hypothesis that practising intubation on a model in multiple configurations would enhance a student’s ability to transfer learned technical skills to laryngoscopy on a new model. Medical and paramedic students with minimal previous laryngoscopy experience trained on one of three models: a novel adjustable manikin in multiple configurations, the same model maintained in a single anatomical position or a commercial non-adjustable manikin.

Students attended a 15-minute didactic session that reviewed airway anatomy, discussed general principles of airway management and explained the procedure for intubation. They watched a short film illustrating intubation and observed a demonstration of the procedure on a manikin. Following this, students were sequentially allocated to one of the three experimental groups. The Laerdal group practised with the Laerdal adult intubation model. The static group used the novel laryngoscopy simulator maintained in the standard configuration (normal face and jaw length, normal dentition and normal head and spine range of motion). The variable group practised on the laryngoscopy simulator, changing the anatomy after every five intubation attempts. The first configuration was standard except that the teeth were removed. Subsequent changes were to replace the teeth, lengthen the face to 0.5 cm more than normal, shorten the mandible by 0.5 cm and finally increase the tension on the mandible slider to adjust the position of the mandible in relation to the skeletal base.

Regardless of group, each subject attempted laryngoscopy with a Macintosh size 3 laryngoscope and intubation with a styletted 7.0 endotracheal tube, 25 times. An investigator observed and scored the result of every attempt as success or failure. After training on the group-specific manikin, all students attempted to intubate the adjustable model with the mouth opening reduced from 5 cm to 3.5 cm, a new configuration for all subjects. In addition, participants performed laryngoscopy with a different airway manikin that none had seen, a Medical Plastics Airway model. Five attempts were recorded on each of the two evaluation models. Success or failure for each attempt was assessed as in the training period.

The authors conducted the analysis by constructing a mixed linear model to take account of the hierarchical structure of the design; multiple measurements at different occasions were nested within each subject.

#### Paramedic self-efficacy and skill retention in pediatric airway management

The aim of the study by Youngquist et al. (2008) was to evaluate the effect of paediatric airway management training and periodic retraining on the self-efficacy and skill performance of paramedics. They hypothesised that an increase in both self-efficacy and skill performance would occur with both training and retraining and that the nature of the retraining programme would have a statistically significant influence on both self-efficacy and skill performance.

This was a prospective, unblinded trial comparing three alternative retraining methods for paediatric airway management with a bag-valve-mask (BVM) and tracheal intubation. A convenience sample of 245 paramedics drawn from the Paediatric Airway Management Project, a paediatric airway management skills course that trained 2520 paramedics from two US EMS services, made up the participants for the study. They were allocated to one of four retraining groups:

1. No retraining (control group).
2. A videotape demonstrating BVM and intubation skills (the same training tape used to supplement initial training (videotape group)).
3. A self-directed learning method, in which paramedics were given instructional materials regarding BVM and intubation skills. Paramedics were encouraged to practise skills in teams, using provided manikins and equipment (self-directed group).
4. A lecture and demonstration by two instructors in addition to instructor-facilitated practice (lecture/demonstration group).

Subjects were asked to complete a 24-question self-efficacy questionnaire both before and immediately after initial training in paediatric airway management (and prior to testing), and again before and after the retraining session. Following completion of all questionnaires, paramedics underwent paediatric airway management skills testing with the use of BVM and paediatric intubation on a manikin. Performance was rated as fail, pass, high pass or honours for each skill. Both training and retraining were performed by the same two research nurses, who also scored paramedics on skills testing based on a written list of skill components. Passing required at least 70% completion of these skill components, high pass required at least 80% and honours was awarded to those successfully completing at least 90% of the skill components.

Chi-square and Fisher’s exact tests were performed to compare categorical variables. Generalised linear models and t-tests were used to test differences in means of normally distributed continuous variables. Kruskal-Wallis tests were used to test for differences in location between non-normally distributed continuous variables. Finally, an ordinal logistic regression model was created to describe the effects of the retraining method on scores obtained in airway skills testing.

## Discussion

The two key review questions that this RER aimed to answer were:

1. How do paramedics learn the skill of intubation?
2. How do paramedics maintain the skill of intubation?

In order to answer these questions, it is first necessary to define when a paramedic is considered to have ’learnt’ the skill of intubation. Although all papers described various methods of learning intubation, including didactic lectures, manikin practice and live patient experience, such as in operating theatres, emergency departments or out-of-hospital, few acknowledge that a definition of having learnt to intubate is not clearly defined.

### Defining intubation skill proficiency/competency

Proficiency and competency were used interchangeably within several studies in this review, and authors did not always clearly define the terms, but all made an association with intubation success rates and/or complication rates as measures of becoming proficient/competent at intubation. Deakin et al. (2009) acknowledged that it was difficult to define the meaning of the word competent in relation to intubation, but intimated that intubation success is a measure of this. Wang et al. (2005) went further, stating that the number of intubation attempts does not demonstrate the quality of performance. While they did define intubation proficiency in terms of the number of successful intubations, they also highlighted that avoidance of technical errors, students’ laryngoscopy technique and sound decision-making should also be in the mix, although admitted that this blend has not been defined. Warner et al. (2010) specifically examined first attempt success at intubation as their primary outcome variable as they felt this was also a measure of minimising potential patient complications.

If intubation success rates (either overall or first attempt) and complication rates are to be included in a definition of having successfully learnt the skill of intubation, then the acceptable percentage of both these rates needs to be decided. Warner et al. (2010) defined a pre-hospital intubation success rate of 90% as an appropriate target for the paramedic students in their study, and this figure appeared in two other studies, although this was not explicitly endorsed as a desirable target (Toda et al., 2013; Wang et al., 2005).

Only Toda et al. (2013) provided any data about complications rates and these arguably have limited applicability since not all of them could be attributable to the intubation attempt. In anaesthetised patients in the operating theatre, they reported a complication rate of 53% decreasing to 31% during the 30 intubation attempts the paramedic students made. The vast majority of complications were minor, either hoarseness or a sore throat, which are not likely to be of great concern to patients eligible for pre-hospital intubation.

### The learning curve for intubation

Three papers aimed to model the relationship between the number of intubation attempts and intubation success (Toda et al., 2013; Wang et al., 2005; Warner et al., 2010). Toda et al. (2013) examined 960 intubation attempts by 32 Japanese paramedics. Having modelled the data, they determined that 31.5 attempts were required for an overall 90% intubation success rate (i.e. all attempts, not just first) with a 95% CI ranging from 27.6 to 54.3 attempts. In addition, the relationship between probability of success and the cumulative number of attempts was S-shaped, with a plateau in intubation success rates for the first 13 attempts, suggesting limited learning, before a sharp rise in success rates at around 19 intubation attempts. However, care needs to be taken when extrapolating models beyond the range of the data provided, and it is not clear how similar Japanese paramedics are to UK paramedic students. Finally, all these intubations took place in the operating theatre and were chosen by the supervising anaesthetist, which might not reflect the challenges of pre-hospital intubation attempts.

This difference in location where the intubation was performed was incorporated into the analysis of the Warner et al. (2010) and Wang et al. (2005) studies. Warner et al. (2010) found a significant, positive relationship between cumulative intubation attempts. In contrast to Toda et al. (2013), overall success rates plateaued at around 10–15 intubations, although the starting success rate in this cohort was above 80%, compared to Toda et al.’s (2013) paramedics with a success rate of around 70%. However, when only first attempt success was examined, intubation success rates started at around 60%, rising to approximately 80% after 20 attempts, leading the authors to conclude that more than 20 attempts would be required to obtain a first-attempt intubation success rate of 90%.

In contrast to the other two studies in this section, Wang et al. (2005) conducted a retrospective review of Fisdap; an internet-based system used to record paramedic student clinical and procedural experience. While not interventional, it did allow the authors to extend their scope beyond a single centre. They included 7635 intubation attempts from 802 paramedic students on 60 paramedic programmes across North America. Like Warner et al. (2010), they stratified their results in order to model intubation success rates by location, and found a significant relationship between cumulative intubation attempts and probability of intubation success. Overall, intubation success rates increased from 77.8% to 95.8% over 30 intubation attempts. However, when examining pre-hospital intubations, success rates started at around 50%, rising to in excess of 85% after 30 attempts. They drew the conclusion that more than 15–25 live intubations, preferably in a range of settings, were required to reach acceptable intubation success rates.

### Intubation training aids

Two papers in the review examined the use of different manikin types as part of paramedic airway education (Plummer & Owen, 2001; Wong et al., 2011); one examined the use of videos from the point of view of a laryngoscopist as part of the students’ training (Levitan et al., 2001), and one aimed to determine whether training intubations in the operating theatre could be replaced by training on a high-fidelity simulator (Hall et al., 2005).

Plummer and Owen (2001) used six different airway trainers (one had two configurations, with and without a cervical collar applied, making seven different airway trainers overall). They found that the students’ success rate was the log of the number of intubation attempts, with an initial steep learning curve that plateaued after approximately 12 attempts. However, changing the airway trainer reduced the odds of a successful intubation by 50%. Only 13 of the 100 students were paramedics and it appeared that the instructor facilitating the event had an impact on intubation success. In addition, the allocation of airway trainer was not consistent, making these results less reliable and applicable to paramedics.

Wong et al. (2011) also tested multiple airway trainers. Their motivation was in part due to the difficulty in obtaining live patient experiences for non-anaesthetists as well as recognising that training on a single model does not translate to good performance on different manikins (or real patients). They developed a novel airway trainer with adjustable anatomy. As with the Plummer and Owen (2001) study, not all of the students were paramedics, although most were (51 paramedic students and 18 medical students). On modelling the data, they too noticed a decline (although a more modest 30% reduction) in intubation success when changing airway trainer.

Levitan et al. (2001) introduced a video showing 15 laryngoscopies from the point of view of the laryngoscopist, which, at the turn of the millennium, was probably very novel. However, these videos are now easy to find on social media, but nonetheless compared to the control group, who only had simple line drawings and manikin anatomy, this appeared to be an advantage when the students went to operating theatres to practise intubation. The mean success rate for the video group was 88.1% (95% CI, 79.6–96.5%) compared to the control group (no video) mean of 46.7% (95% CI, 42.2–51.3%). This study did use historic controls, so it is possible that other confounding factors have not been taken into account.

Given the limited opportunities to gain intubation experience in operating theatres for paramedic students (Deakin et al., 2009; Johnston et al., 2006) both in the UK and the US, it is no surprise that a study has been conducted to see whether some of these intubation attempts on live patients could be replaced by high-fidelity simulation. Hall et al. (2005) conducted an RCT with a group of 42 paramedic students. Having completed the standard airway training curriculum consisting of 20 hours of didactic and video training, supplemented with 10 hours of practice on a manikin, students were randomised into either receiving 10 hours of training on a high-fidelity simulator (METIman) facilitated by an anaesthetist and senior emergency department doctor, or being in the control group and going to theatres to perform 15 intubation attempts. Students in the intervention arm (simulation group) completed an ’average’ of 50 intubations on the simulator. Following this, both groups were assessed in the operating theatre by performing 15 intubation attempts. The first-attempt success rates were 84.4% and 80.0% in the simulation and control group, respectively, which was a non-significant difference. This led to the conclusion that paramedic students could be trained on high-fidelity simulators as effectively as traditional live patient intubation when assessed in the operating theatre. While Hall et al. (2005) do acknowledge the cost of the manikin as an issue, the possibility that the experience of the faculty also contributed to the performance of the simulator group was not considered.

### Maintaining the skill of intubation

Compared to skill acquisition, there was a paucity of evidence that specifically addressed how paramedics maintained proficiency/competence in the skill of intubation. Two papers (Deakin et al., 2009; Youngquist et al., 2008) in the review addressed this issue, although Deakin et al. (2009) conducted a telephone survey of training schools in 2008, which is unlikely to provide more than a historic view of the maintenance of advanced airway skills. The study by Youngquist et al. (2008), however, did examine the performance of paramedics between two time points (a median of 13 months, IQR 7–16 for all groups). Although the study only considered intubation in children, there was a significant decline in skills performance as time to retraining increased (odds ratio (OR) 0.93, 95% CI, 0.87–0.98). As an RCT evaluating different types of training modality (video only, self-directed learning or lecture and demonstration by instructors followed by instructor-facilitated practice), the study also revealed that the only retraining intervention that had a significant increase in skills performance was the lecture/demonstration group (OR 5.17, IQR 2.39–11.19). These results suggest that good quality retraining can help in improving skills performance (at least during an assessment on a paediatric manikin), especially in a patient group where clinical exposure is likely to be limited.

### Limitations

There are a number of important limitations with this review. The most significant is the methodology. Although RERs do emulate aspects of a systematic review, they are not as comprehensive due to time and resource constraints. It is possible that relevant papers were not included in the review. In addition, the RER was conducted by a single person (RP), which may have resulted in papers being excluded from the study inappropriately. Finally, given the limited data to inform the research question, it may have been prudent to have widened the eligible population, although this would have potentially made findings less relevant to paramedics.

## Conclusion

In order to determine how paramedics learn and maintain the skill of intubation, a definition is required so it is clear what benchmark is to be used to state that a paramedic has ’learnt’ the skill of intubation. This could be a single measure such as first-pass success of 90% for pre-hospital intubation, for example, or a range of measures, such as intubation success and complication rates, laryngoscopy technique and decision-making.

The precise number of intubations required to become proficient at intubation is not clear, but based on the evidence in the review, to achieve a first-pass intubation success rate of 90%, paramedic students require 25–30 intubations on live patients, preferably in a range of environments (e.g. commencing in operating theatres or other in-hospital settings and then out-of-hospital). Additional intubations are desirable, either with live patients or on high-fidelity manikins.

Intubation training should use a range of modalities, including didactic lectures, videos and practical sessions on airway manikins. Students should not be trained on only a single manikin, but should have access to multiple types. Supervision by experienced faculty is required.

Little is known about how paramedics maintain their skill in intubation, given the lack of clinical opportunity. Yearly skills retraining can help, and can be enhanced by demonstrations/lectures from experienced faculty.

Further research is needed to understand how para-medics maintain their skill in intubation, given the limited opportunities to use the skill in a clinical setting and lack of opportunities with UK ambulance services for retraining.

## Acknowledgements

Thanks to Professor Julia Williams from the College of Paramedics’ Airway Working Group for her assistance in developing the protocol for this rapid evidence review. In addition, the support from the librarian at the University of Hertfordshire, who provided some early guidance on potential search terms and MESH headings for the literature review, also deserves recognition.

## Conflict of interest

Richard Pilbery is the Editor of the *British Paramedic Journal*.

## Funding

## This report was commissioned and paid for by the College of Paramedics.
